# Modern Methods in War from a Medical Standpoint

**Published:** 1919

**Authors:** 


***Modern Methods in War from a Medical Standpoint.*** By Surgeon-General J. T. FOTHERINGHAM, C. M. G. *The Proceedings of the Institute of Medicine of Chicago*, November 2, 1917.

The author gives a brief description of the organization of the Canadian Medical Service and a history of the Second Division, thus illustrating the work carried out.

He further speaks of gas attacks and of the manner in which the effects of hydrocyanic and mustard gas were mastered, quoting a report of the British Medical Journal of October 13, 1917 on the activities of the C. A. M. C.

With regard to trench foot, the author's observations may be summarized as follows. The 3 essentials of trench foot are : wet, temperature from 20 to 40 F., and infection. Cases may be classified according to severity into 3 groups : mild cases, recovering in about a week but leaving a tendency to relapse; moderate, requiring at least a month; severe, requiring 6 months and more. The occurrence of trench foot was great during the first year of the war, but it subsided as soon as it was made a matter of discipline.

Sick-wastage has been comparatively small in the Canadian forces, thanks to the special care taken in that respect. The following points, mainly, were emphasized :

1. Securing all possible rest for the men.
2. Controlling the feeding of the men : hot food for working parties, etc.
3. Attention to regular use of bathing facilities.
4. Doing everything possible to have proper water available.
5. All points bearing on the men's health were daily insisted on by the M. O.

Sanitary inspections were made at least once in 48 hours, and special attention was paid to latrines, food waste, water discipline, vermin and flies, stagnant water in trenches, etc.

The author speaks of the services rendered by M. C. A.'s and insists upon the value of horse ambulances which go where motor ambulances cannot go, across fields, etc., and are able to proceed without damage at the pace of a moving infantry column.

The work and duties of the M. O. are further recorded. The main considerations in that respect are the following : lying and walking cases should never be handled together, nor brought to the same dressing stations; separate arrangements must be made for the various categories of wounded.

